# Proposal of a Plan for the Investigation of the Due Administration of Blood-Letting

**Published:** 1830-01-01

**Authors:** 


### PROPOSAL OF A PLAN FOR THE INVESTIGATION OF THE DUE ADMINISTRATION OF BLOOD-LETTING. By *Marshall Hall,* M.D. &c.

WE learn from this little brochure that Dr. Hall is prosecuting with his usual zeal, and, we have no doubt, with his usual ability, the very important subject of blood-letting. No remedy is now more general—not even calomel or blue-pill—and this fact may assure us that no remedy is more abused. It is certain that if several persons be bled to deliquium in the erect posture, they being of apparently similar strength, but affected with dissimilar diseases, they will be found to have lost very different quantities of blood before the fainting is induced. Dr. Hall has known a patient, not apparently very feeble, to faint on losing four ounces of blood—whilst he has seen other patients bear the loss of 50, 60, or even 70 ounces of blood without syncope. How is this to be explained?

"Its rationale is to be found, I believe in connection with an equally interesting fact, that different diseases induce in the constitution different powers or susceptibilities in regard to the effects of loss of blood. Each disease appears, indeed, to possess its own peculiar and intrinsic virtue in this respect. This is determined by placing the patient perfectly erect, and bleeding to incipient syncope; the quantity of blood which flows is the measure of the protective influence of the disease in one class of cases, and of its influence in superinducing a susceptibility to the effects of loss of blood in the other.

"An interesting scale of diseases may be formed representing these properties. It would begin with congestion of the head, or tendency to apoplexy; inflammation of the serous membranes, and of the parenchymatous substance of various organs, would follow; and lastly, inflammation of the mucous membranes. This part of the scale would be divided from the next by the condition of the system in health. Below this would be arranged fever, the effects of intestinal irritation, some cases of delirium, reaction from loss of blood, and disorders of the same class with hysteria, dyspepsia, chlorosis, and cholera morbus." 4.

Persons in health and of moderate strength, will generally faint if bled in the erect posture on taking fifteen ounces of blood. Dr. H. has known seventy ounces to be taken in the sitting posture, in tendency to apoplexy, without syncope. But this is an extreme case. Patients with pleuritis frequently bear the loss of 35 ounces of blood without fainting. "In bronchitis little more is borne to be lost than in health."

"A stout person in fever will frequently faint on losing ten, twelve, or fourteen ounces of blood. In intestinal irritation, with urgent symptoms even, the abstraction of nine or ten ounces of blood will generally induce deliquium. In delirium tremens, or puerperal delirium, the patient soon faints from loss of blood. The same thing is still more observed in those cases of violent reaction, which arise from loss of blood itself. In dyspepsia, hysteria, and chlorosis, the susceptibility to syncope from loss of blood is very great. And I have known a patient, of good strength, affected with cholera, faint on taking four ounces of blood, although she had shortly before borne to lose nearly twenty ounces without faintishness, under the influence of inflamed mamma." 5.

The solution of these differences, Dr. H. thinks, must be found in the different nature of the diseases themselves. In all those cases, he observes, where the circulation of the heart and larger arteries only is affected, and especially in such as involve irritation or exhaustion, there is early syncope on taking blood. But in such cases as consist in an affection of the capillary circulation, and especially such of these as affect the head, it requires the abstraction of much blood to induce deliquium.

"Syncope is prevented by the influence exerted by this state of the capillary circulation over that of the heart and larger arteries, and over the whole system, and especially over the circulation within the brain; and it does not entirely subdue the morbid action of the capillary vessels even when induced. To induce syncope in pure fever, we have then but to subdue the state of reaction in the heart and larger arteries. In inflammation, we have not only to do this, but to overcome the influence of a permanent morbid action of the capillaries; this is especially observed in inflammation of the serous membranes and within the head." 6.

The practical application of this fact consists chiefly in its affording a rule for blood-letting in all cases in which this measure is required to be fully instituted—a guard against undue blood-letting, both in this and some other cases—and a source of diagnosis.

"The quantity of blood which flows when a patient requiring full blood-letting is placed upright and bled to deliquium, seems accurately proportionate to the exigencies of the case. In inflammation much blood should be taken; and much blood will flow before deliquium is induced: in irritation, little blood should be drawn; and there is early syncope from blood-letting. The quantities are even accurately suited, not only to the exigencies of the disease, but to the powers of the system; at least so it appears to me from considerable experience.

"The rule is suited also to the degree and the duration of the disease, for, with each of these, its influence in inducing tolerance or intolerance of loss of blood is respectively augmented.

"It is not less adapted to those most frequent of all events, mixed cases. Inflammation and irritation may be conjoined. For example, there may be mere nephralgia, or absolute nephritis, from calculus, or a mixed case involving both. There may be mingled intestinal irritation, and inflammation. In each of these circumstances, the rule for blood-letting which I have proposed, adapts itself accurately to the demands of these various morbid affections, and to the actual strength and condition of the general system.

"It is difficult to say whether more injury has been done by an undue or by an inefficient use of the lancet. In inflammation we must bleed fully. In irritation we must bleed cautiously. Inefficient blood-letting in the former disease, and undue blood-letting in the latter, are alike dangerous or even fatal to the patient; from both extremes we are guarded by the rule which I propose. By directing the patient to be placed in the erect position, and bled to deliquium, we often take much more blood than we should have ventured to prescribe, in inflammation, and very much less than we might be disposed to direct, in irritation; and in both these cases the rule conducts us to the only safe mode of treatment.

"A further practical application of this fact, flows from the adoption of the rule. In doubtful cases it furnishes us with a fresh means of diagnosis. If much blood has flowed before syncope occurred, we must suspect inflammation; if little, we must suspect that, however similar the symptoms, the case is in fact of a different nature,—perhaps irritation, perhaps exhaustion. For further information on these two states and affections of the system, I must refer my reader to a little work in the press, entitled Researches on the Morbid and

Curative Effects of Loss of Blood, in which they will be particularly discussed." 9.

The principal object of this precursor of a larger work is to solicit the co-operation of the author's medical friends in the further investigation of the subject. With the view of obtaining facts, by the multitude of which alone the propositions can be established or corrected, he proposes that, in every case in which full blood-letting is to be instituted, the patient should be placed perfectly erect in a chair or in bed, and bled to the very first appearance of syncope. The quantity of blood taken is then to be noted, and accurately registered in a table. The same thing is to be observed on each repetition of the bleeding. The various facts which he proposes to register may be seen in the following table.

| Age and strength of the patient. | Disease, its stage & complications. | Quantity of blood taken. | Effects on the patient and disease. | Appearances of the blood. | Repetitions of the blood-letting. | Effects. |
|---|---|---|---|---|---|---|
| | | | | | | |

Dr. H. observes, in conclusion, that he does not think it safe, in any case, to bleed to deliquium in the recumbent posture. There can be few, if any cases, in which, if it be proper to bleed fully, danger can accrue from bleeding to the most incipient syncope in the perfectly upright position. Besides, the remedy is at hand. We have only to lay the patient in the recumbent posture, and, if necessary, raise the feet and depress the head.

We shall be anxious to see the promised work, announced by the talented author in one of the foregoing extracts.

